# Comparative Study of Wear Behavior of Hypereutectic Al–Si Piston Alloys Using Experimental and Numerical Methods

**DOI:** 10.3390/ma19112253

**Published:** 2026-05-26

**Authors:** Atanasi Tashev, Valyo Nikolov, Boyan Dochev, Desislava Dimova, Mara Kandeva, Mihail Zagorski

**Affiliations:** 1 Department of Transport and Aircraft Equipment and Technologies, Faculty of Mechanical Engineering, Technical University of Sofia, Branch Plovdiv, 25 Tsanko Dyustabanov Street, 4000 Plovdiv, Bulgaria; vnikolov@tu-plovdiv.bg; 2 Center of Competence “Smart Mechatronic, Eco- and Energy-Saving Systems and Technologies”, 4000 Plovdiv, Bulgaria; dochev@tu-plovdiv.bg; 3 Department of Mechanical Engineering and Technologies, Faculty of Mechanical Engineering, Technical University of Sofia, Branch Plovdiv, 4000 Plovdiv, Bulgaria; 4 Department of Theory of Mechanisms and Machines, Faculty of Industrial Technology, Technical University of Sofia, Branch Plovdiv, 1756 Sofia, Bulgaria; kandevam@gmail.com (M.K.); mzagorski@tu-sofia.bg (M.Z.)

**Keywords:** hypereutectic Al–Si alloys, piston materials, wear resistance, tribology, Archard model, finite element analysis, contact pressure

## Abstract

This study presents an integrated experimental–numerical approach for evaluating the wear behavior of three non-standardized hypereutectic aluminum–silicon (Al–Si) piston alloys based on the AlSi25CuCr system, namely AlSi25Cu4Cr (M1), AlSi25Cu5Cr (M3), and AlSi25Cu5Cr (M5). The wear coefficient was determined experimentally under boundary-lubrication conditions, while the contact conditions in the piston–cylinder system were evaluated using Finite Element Analysis (FEA) and implemented within the Archard wear model. The results reveal a pronounced inconsistency between hardness and wear resistance. Although hardness increases from 1363 MPa (M1) to 1677 MPa (M5), the corresponding wear depth increases from 13.94 nm to 27.61 nm per engine cycle. This behavior is attributed to differences in microstructural characteristics, particularly the morphology and distribution of silicon particles and intermetallic phases, which significantly influence the tribological performance of hypereutectic Al–Si alloys. The experimentally determined wear coefficient K also shows a significant increase, rising from 12.14 × 10^−5^ (M1) to 29.59 × 10^−5^ (M5). The lowest wear is observed for alloy M1, whereas M5 exhibits the poorest tribological performance. These findings demonstrate that microstructural characteristics, particularly the morphology and distribution of silicon particles and intermetallic phases, have a dominant influence over hardness in governing wear behavior. The main scientific contribution lies in the direct coupling of experimentally determined material properties with realistically simulated contact conditions, enabling a quantitative and physically consistent comparison of piston alloys under identical operating regimes. The proposed methodology provides a reliable framework for material selection and optimization of piston alloys with enhanced wear resistance.

## 1. Introduction

Internal combustion engines (ICEs) transform the thermal energy released during fuel combustion into mechanical work via the crankshaft [1]. They remain a key technology in transportation and distributed power generation due to their high specific energy, robustness, and well-established manufacturing ecosystem. Despite the growing transition toward electrification, a large portion of global mobility continues to rely on ICE-based systems, particularly in commercial transport and hybrid applications. This sustained relevance drives ongoing research aimed at improving efficiency, reducing emissions, and extending component service life [2,3,4,5,6,7,8,9,10,11,12,13]. At the same time, the continuous increase in the number of vehicles [14,15] worldwide intensifies the demand for durable and reliable engine components capable of operating under progressively stricter conditions.

In this context, tribological performance has emerged as a critical factor governing both efficiency and durability of internal combustion engines [16,17,18]. A significant portion of engine losses originates from friction within moving assemblies, among which the piston–ring–cylinder system represents one of the most heavily loaded and tribologically complex subsystems. Beyond energy losses, wear phenomena in this system directly influence oil consumption, sealing effectiveness, noise generation, and emission characteristics. Consequently, minimizing wear while maintaining stable friction behavior is essential for improving both environmental and operational performance of engines.

The piston plays a central role in this system, acting as the interface between the combustion process and the mechanical power transmission chain. During operation, it is subjected not only to high-pressure gas forces and inertial loads but also to repeated lateral contact with the cylinder liner, driven by the geometry of the crank–connecting rod mechanism. This interaction results in a cyclic variation in contact conditions along the piston stroke, where hydrodynamic lubrication is not always maintained. Particularly in the vicinity of the top and bottom dead centers, the combination of low sliding speed and high normal load leads to mixed or boundary lubrication regimes, significantly increasing the likelihood of wear.

Under these conditions, the piston skirt and ring belt regions experience complex and evolving wear processes. Material degradation may occur through a combination of adhesive transfer, abrasion by hard asperities or particles, oxidative mechanisms at elevated temperatures, and fatigue-induced surface damage. The dominance of a given wear mechanism depends on operating parameters such as contact pressure, temperature, lubrication regime, and surface topography, as well as intrinsic material properties such as hardness, microstructure, and phase composition. Over time, these processes lead to measurable material loss, modification of surface geometry, and deterioration of functional performance.

The consequences of wear in the piston assembly are multifaceted. Progressive material removal from the skirt alters the guiding function of the piston, potentially leading to increased clearances, vibration, and acoustic emissions. Simultaneously, wear in the ring zone affects sealing efficiency, which may result in blow-by, increased oil consumption, and higher emissions. From a system perspective, even relatively small changes in wear rate can significantly influence engine lifetime and maintenance intervals. Therefore, improving the wear resistance of piston materials remains a central objective in engine design and materials engineering.

Among the materials used for piston manufacturing, aluminum–silicon (Al–Si) alloys occupy a dominant position due to their advantageous combination of low density, high thermal conductivity, and excellent castability [19,20,21,22]. These properties enable a reduction in reciprocating mass and efficient heat dissipation, which are essential for high-speed engine operation. At the same time, the tribological behavior of Al–Si alloys is strongly dependent on their microstructural characteristics, particularly the distribution, size, and morphology of free silicon crystals and intermetallic phases [23,24,25,26,27].

Hypereutectic Al–Si alloys are characterized by high wear resistance. The presence of hard silicon crystals embedded in a relatively soft aluminum matrix results in a composite-like microstructure that provides an effective strengthening mechanism and enhances resistance to surface damage under sliding conditions [23,24,25,26,27]. In addition, the relatively low coefficient of thermal expansion contributes to improved dimensional stability at elevated temperatures, which is essential for maintaining optimal clearances in the piston–cylinder system. However, the effectiveness of this mechanism strongly depends on the degree of microstructural modification. Coarse or irregular silicon crystals may act as stress concentrators, leading to crack initiation and deterioration of both fatigue strength and wear resistance [28,29,30].

Various alloying and processing strategies have been developed to optimize the performance of Al–Si piston alloys. The addition of elements such as Cu, Ni, Mg, Cr, and Mn promotes the formation of thermally stable intermetallic phases, enhancing strength and resistance to high-temperature degradation [22,28,29,30,31,32]. Heat treatment (T6) further improves the distribution of strengthening phases, resulting in enhanced mechanical and tribological properties [33,34,35,36]. In parallel, modification techniques aimed at refining primary silicon, such as the addition of phosphorus or rare earth elements, contribute to the formation of a finer and more uniform microstructure, thereby improving wear resistance [23,24,25,26,27].

Recent studies have also focused on advanced approaches for microstructural refinement and predictive modelling of aluminum-based materials. The application of grain refiners such as Al–5Ti–0.2C has demonstrated significant improvement in the microstructure and mechanical properties of Al–Si–Mg–Cu alloys through enhanced grain refinement and phase distribution control [37]. In parallel, integrated experimental and computational approaches, including intelligent modelling techniques, have been applied for analysing the mechanical behaviour of aluminum-based functionally graded materials under complex operating conditions [38]. These investigations confirm the increasing importance of combining microstructural optimization with advanced predictive methodologies for the development of high-performance aluminum alloys.

Although the structural and thermal behavior of Al–Si alloys has been extensively studied [20,21,39,40,41,42,43,44], their tribological performance is often addressed in a limited or indirect manner. This necessitates the development of more comprehensive approaches that explicitly account for wear mechanisms and their dependence on material properties and operating conditions.

The integration of experimental characterization with computational modeling has opened new possibilities for analyzing wear processes. Approaches based on contact mechanics and the Archard wear model enable quantitative evaluation of material removal as a function of contact pressure, sliding distance, and material hardness [18,43,44]. When combined with experimental data, these methods provide a reliable framework for comparing materials under controlled conditions.

As can be seen from Table 1, previous studies mainly focus on thermal-structural analysis or experimental tribological characterization separately. However, limited attention has been paid to the combined evaluation of experimentally determined wear coefficients and theoretical wear prediction of non-standardized piston Al-Si alloys under engine-representative loading conditions. Therefore, the aim of the present study is to addresses this gap by investigating three hypereutectic alloys (M1, M3, and M5) using a combined approach that integrates experimental determination of material properties with numerical modeling of contact conditions in the piston–cylinder system.

The scientific contribution of this work lies in the direct coupling of experimentally measured material properties with a wear model, enabling a physically consistent comparison of materials under identical operating conditions. Furthermore, the study contributes to a deeper understanding of the relationship between composition, microstructure, and wear resistance, providing practical guidelines for the optimization of piston materials and offering potential for application to other tribological systems in internal combustion engines.

## 2. Materials and Methods

The investigated materials are hypereutectic aluminum–silicon alloys from the AlSi25CuCr system, namely AlSi25Cu4Cr (M1) and AlSi25Cu5Cr (M3). The alloys are additionally alloyed with refractory elements (Ni, Co, and Mo), as well as their various combinations. For comparative purposes, the AlSi25Cu5Cr alloy (M5), which does not contain additional refractory elements, was also included. The detailed chemical composition and processing route of the alloys are presented in previous studies [45,46]. A summary of the principal chemical and mechanical properties of the investigated alloys is presented in Table 2 and Table 3.

The investigated alloy system was selected based on the widespread application of hypereutectic Al–Si–Cu alloys in highly loaded internal combustion engine pistons, where high wear resistance, thermal stability, and dimensional stability at elevated temperatures are required. The AlSi25Cu4Cr and AlSi25Cu5Cr base compositions were chosen due to their high silicon content, providing enhanced tribological performance, while Cu and Cr contribute to strengthening and thermal resistance through the formation of stable intermetallic phases. Additional alloying with refractory elements such as Ni, Co, and Mo was introduced in order to investigate their influence on phase composition, microstructural stability, and wear behavior. These elements are known to promote the formation of thermally stable intermetallic compounds and to influence silicon morphology and high-temperature performance of piston alloys.

All alloys were modified with phosphorus, with a content of 0.04 wt.% for alloy M1 and 0.07 wt.% for alloys M3 and M5. After casting, the specimens were subjected to T6 heat treatment. Homogenization was carried out at 510–515 °C with a holding time of 6 h 30 min, followed by water quenching at 20 °C. Artificial aging was performed at 180 °C for 12 h. The mechanical properties were determined experimentally and are described in detail in [45,46].

In the present study, these materials were used for the analysis of tribological behavior and wear under controlled conditions.

The evaluation of piston wear was performed based on the classical Archard wear equation [16,18,47,48]:

(1) $$h=\frac{K\times p\times s}{H}$$

where *h* [mm] is the wear depth, *K* [–] is the wear coefficient, *p* [MPa] is the contact pressure, *s* [mm] is the sliding distance, and *H* [MPa] is the material hardness.

According to Equation (1), the determination of wear requires independent evaluation of the wear coefficient *K*, the contact pressure *p*, the sliding distance *s,* and the material hardness *H*.

### 2.1. Determining Wear Coefficient

**Figure 1:**
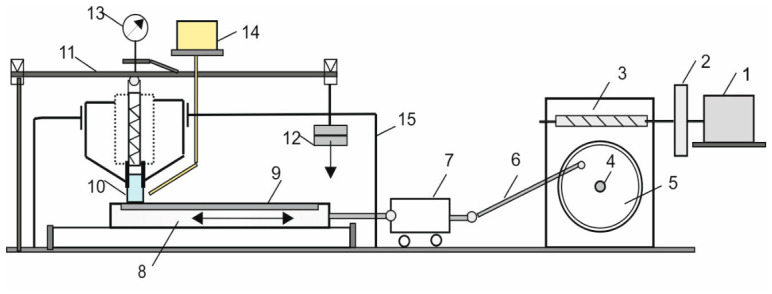
Device RT16 for study of wear at reversive motion.

The wear behavior of the contact system (tribosystem) was investigated under boundary lubrication conditions using an RT16 tribotester (Technical University of Sofia, Sofia, Bulgaria) operating in a reciprocating sliding mode. A schematic representation of the experimental setup is shown in Figure 1. The contact was established between a vertically mounted cylindrical specimen (10) and a horizontally positioned counterbody (9). Three different materials (M1, M3, and M5) were examined. The counterbody (9) was fixed onto a carriage (8), which performed a reciprocating motion.

The motion was generated by an electric motor (1), with torque transmitted via a coupling (2) to a worm gearbox (3). A disc (5), mounted on the gearbox output shaft (4), drove a crank-slider mechanism. The connecting rod (6) was pivotally connected to a slider (7), which transferred reciprocating motion to the carriage (8) and the attached counterbody (9). The design of the carriage system, including the articulated joint and cylindrical guide rod, ensured stable bidirectional motion through self-alignment of the contacting bodies. This configuration minimized parasitic friction and vibrations within the system. The test specimens (10) had a cylindrical geometry with a contact surface diameter of 3 mm. The counterbody (9) was a flat plate with dimensions of 107 mm in length, 43 mm in width, and 2 mm in thickness. The normal load applied to the specimen was controlled using a lever system consisting of a dynamometric beam (11) with a lever ratio of 1:3.2 and calibrated weights (12). The maximum applicable load of the device was 160 N. Wear was monitored using a vertical displacement indicator (13), allowing measurement of the relative height reduction (wear depth) of the specimen with a resolution of 1 µm. Lubrication was provided via a drip lubrication system (14), supplying oil directly to the contact zone at a flow rate of 40 drops per minute. The kinematic parameters of the tribotester were as follows: a reciprocating frequency of 28 cycles/min, resulting in a sliding distance of 3.92 m per minute (calculated as S=0.14×28) and a sliding velocity of 6.8 cm/s.

The experimental methodology consisted of a series of controlled and repeatable steps. Initially, six identical specimens from each test material and six identical counterbodies were prepared, all having the same surface roughness of the contact interface. The roughness was measured using a TESA Rugosurf 10-10G profilometer (TESA SA, Renens, Switzerland). The initial mass *m*_0_ of each specimen was determined using an electronic balance WPS 180/C/2 with an accuracy of 0.1 mg. Prior to each measurement, the specimens were carefully cleaned of mechanical and organic contaminants and subsequently dried in a drying chamber for 15 min at 150 °C to remove any residual oil from the material pores. Each specimen (10) was rigidly fixed in its holder, while the corresponding counterbody (9) was mounted on the reciprocating carriage (8). A predefined normal load was applied using the calibrated weights (12), and the lubrication system was filled with the test oil. The electric motor (1) was then activated, and the test was conducted for a predefined sliding distance or time. The duration of friction was measured using a stopwatch with an accuracy of 1 s.

After completion of the test, the specimens and counterbodies were removed, cleaned using a degreasing agent, and dried again under the same conditions. The final mass $m_{i}$ was then measured using the same balance. The wear behavior was evaluated based on the mass loss of the specimens and counterbodies, determined as: Δ*m* = *m*_0_ − *m_i_*, where *m*_0_ is the initial mass and *m_i_* is the final mass after the test. The experimentally measured mass loss was first converted into wear volume according to:

(2) $$∆V=\;\frac{∆m}{\rho }$$

where ΔV [mm^3^] is the wear volume, Δm [mg] is the measured mass loss, and ρ [mg/mm^3^] is the material density.

The wear coefficient K used in the Archard equation was then calculated as:

(3) $$K=\frac{∆V\times H}{F\times s}$$

where H [N/mm^2^] is the Vickers hardness converted into MPa, F [N] is the applied normal load, and s [mm] is the sliding distance during the tribological test. This formulation is derived from the Archard relation by expressing the experimental wear depth as h = ΔV/A and the nominal contact pressure as p = F/A. Therefore, the contact area A cancels out, resulting in a dimensionless wear coefficient K suitable for use in Equation (1).

The obtained results were used to calculate the corresponding wear characteristics under constant operating conditions, including applied load, sliding speed, friction distance (or time), and lubricant type. Based on the mass loss, material density and the specimen geometry, the wear factor is calculated for each test material. The test parameters and material properties are presented in Table 4.

### 2.2. Determining Material Hardness

The hardness values used in the present study correspond to Vickers macrohardness measurements (HV5), determined in accordance with ISO 6507-1 [49] under a test load of 5 kgf. The obtained hardness values were expressed in kgf/mm^2^ (HV). To ensure consistency with SI units, the measured hardness values were converted into MPa according to the relation: H [MPa] = HV × 9.807, where HV is the Vickers hardness expressed in kgf/mm^2^. This conversion is required for the application of the Archard model, in which hardness is used as a parameter characterizing the material’s resistance to wear. According to Equation (1), higher hardness corresponds to lower wear depth.

### 2.3. Determining Contact Pressure and Sliding Distance

**Figure 2:**
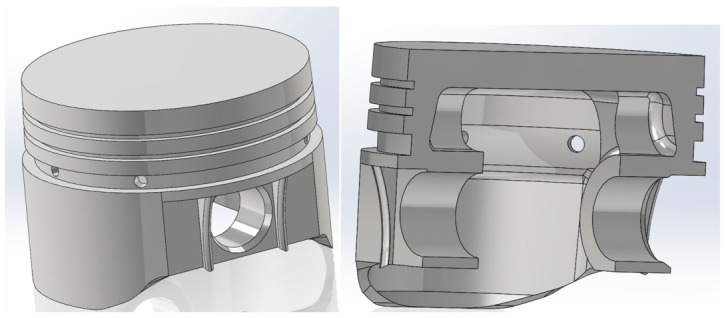
Three-dimensional model of the piston.

A three-dimensional (3D) model of the piston was developed using SolidWorks 2022 SP03.1 (Figure 2). The geometry corresponds to a modern naturally aspirated 1.5 L gasoline engine with a compression ratio of 11:1 and a rated power of 80 kW at 6000 rpm. The piston diameter is 72 mm, and the stroke *S* is 92 mm. These parameters were selected to ensure a realistic representation of operating conditions typical for contemporary spark-ignition engines.

**Figure 3:**
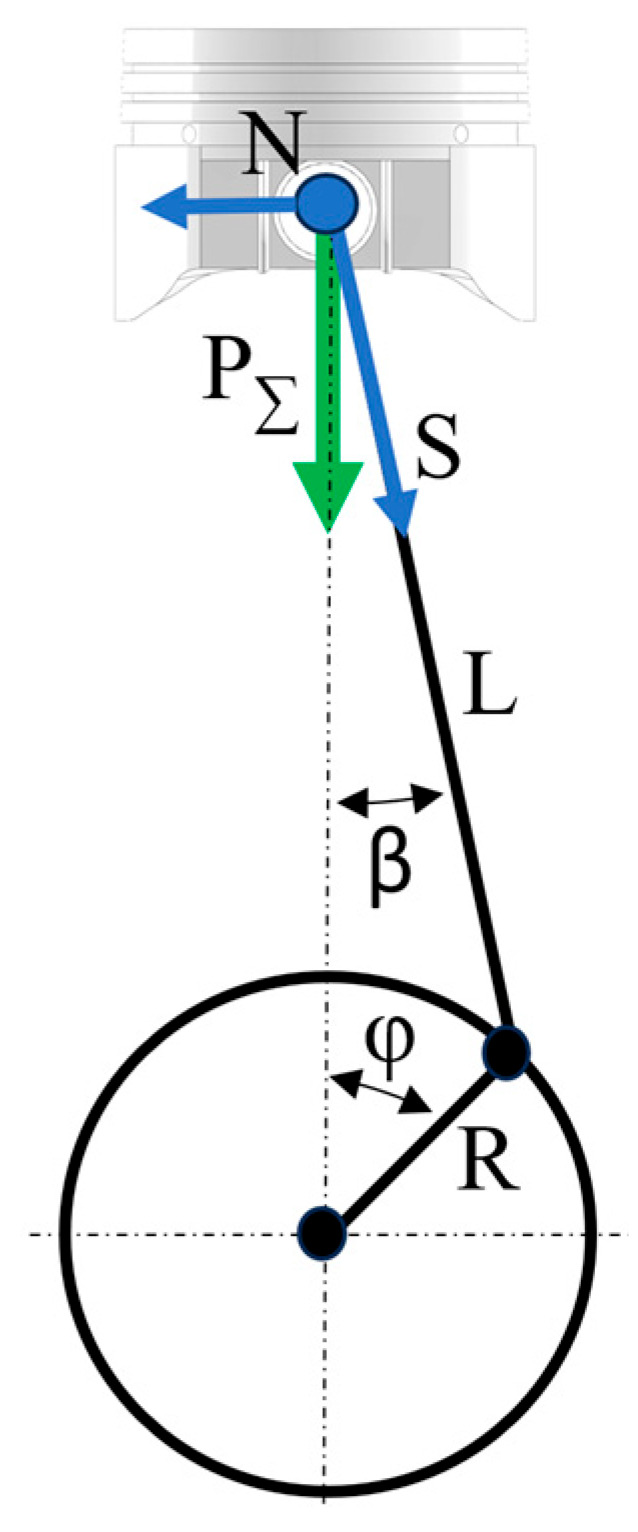
Diagram of a crankshaft mechanism of an ICE with a resultant force *P*_∑_ and a lateral (normal) force *N*.

Based on thermodynamic and dynamic analysis of the engine cycle, the resultant force *P*_∑_, combining gas pressure and inertial effects, was determined as a function of crank angle φ (Figure 3). The lateral (normal) force acting on the piston skirt is expressed as:

(4) $$N=P_{\sum }\tan\beta$$

where βs is the instantaneous angle of the connecting rod relative to the cylinder axis.

The angle *β* is related to the crank angle φ through the kinematic relation:

(5) $$\tan\beta =\frac{R\cdot \sin\varphi }{\sqrt{L^{2}-R^{2}\cdot {sin}^{2}\varphi }}$$

where *R* is the crankshaft radius, and *L* is the connection rod length.

**Figure 4:**
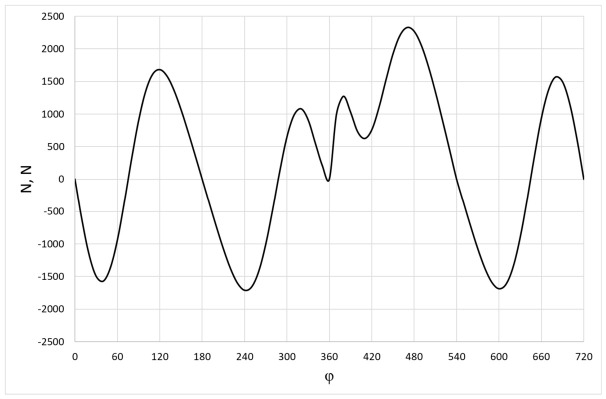
Graph of normal force *N* per one working cycle—two full rotations of the crankshaft.

**Figure 5:**
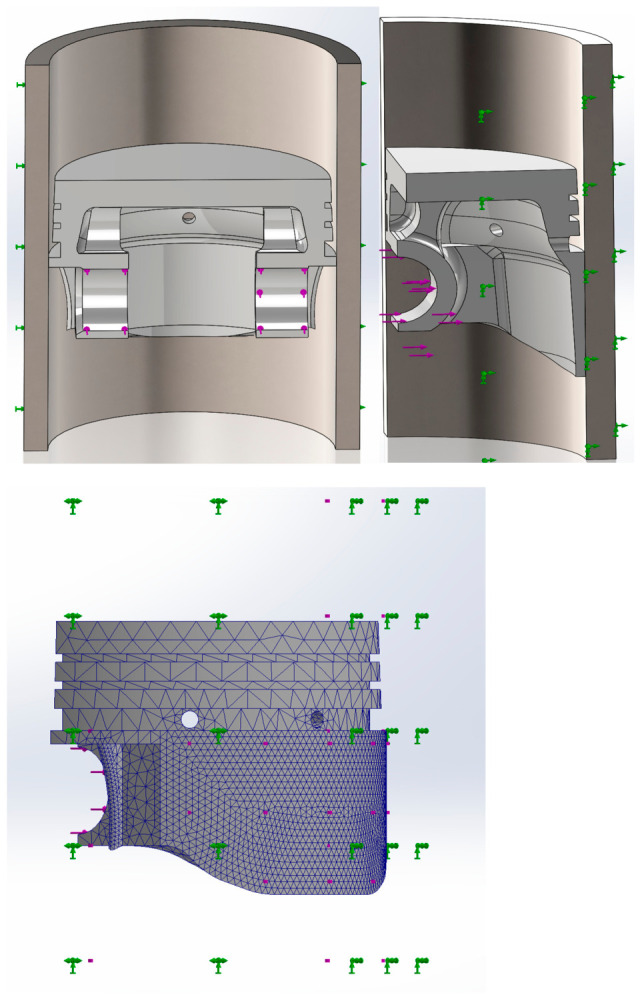
FEA setup.

The calculated normal force profile over one working cycle (720° crank angle), presented in Figure 4, was used as input for a finite element analysis (Figure 5). The simulations were performed under quasi-static conditions, applying the load incrementally to obtain the contact pressure distribution along the piston skirt.

Since the direction of the lateral force alternates during the cycle, only the positive values (corresponding to actual contact with the cylinder liner) were considered in the pressure evaluation.

The sliding distance was defined based on piston kinematics. For one complete engine cycle, the piston travels twice the stroke in each direction, resulting in a total sliding distance: s = 4*S*.

To account for the varying load during the cycle, an equivalent contact parameter was determined by integrating the contact pressure over the crank angle range where contact occurs. This approach allows coupling of the experimentally determined wear coefficient with the numerically obtained contact pressure distribution for wear estimation using Equation (1).

The finite element model was developed to represent the contact interaction between the piston skirt and the cylinder liner. The cylinder was defined as a rigid or high-stiffness body, while the piston was modeled as a deformable body with material properties corresponding to the investigated alloys.

A surface-to-surface contact formulation was applied between the piston skirt and the cylinder liner. A constant friction coefficient of μ = 0.1 was applied in the contact definition between the piston skirt and cylinder liner surfaces. This value was not experimentally determined within the present study but was selected as a representative engineering value for lubricated aluminum–steel contacts operating under boundary lubrication conditions, based on published tribological investigations and typical piston–cylinder friction conditions reported in the literature. Since the primary objective of the numerical analysis was a comparative evaluation between the investigated materials, the same friction coefficient was applied consistently for all simulations.

The piston was constrained to allow motion only along the cylinder axis, while lateral displacement was governed by the applied normal force. The connection region (piston pin bore) was loaded using the calculated force N(φ), applied as a distributed load over the pin contact surface.

The mesh was refined in the piston skirt region to ensure accurate resolution of the contact pressure distribution, while a coarser mesh was used in regions of lower stress gradients. A mesh sensitivity consideration was applied to ensure that the obtained contact pressure results were independent of the mesh density.

The use of area-averaged contact pressure ensures consistency with the Archard wear formulation, where macroscopic contact conditions are considered.

Microstructural characterization and phase analysis were carried out using optical microscopy and X-ray diffraction (XRD), as described in detail in [45,46]. The obtained results were used in the present study to interpret the tribological behavior and wear of the investigated alloys.

## 3. Results and Discussion

### 3.1. Material Properties and Wear Coefficient

The mechanical and tribological properties of the investigated materials are summarized in Table 5. The hardness values range from 1363 MPa (M1) to 1677 MPa (M5), indicating a progressive increase in material resistance to plastic deformation.

At the same time, the experimentally determined wear coefficient *K* shows an opposite trend, increasing from 12.14 × 10^−5^ (M1) to 29.59 × 10^−5^ (M5).

This behavior suggests that although hardness increases, the wear resistance does not improve proportionally. Such a trend indicates that wear behavior is influenced not only by hardness but also by microstructural characteristics, such as the distribution of alloying elements and secondary phases.

### 3.2. Contact Pressure Distribution

**Figure 6:**
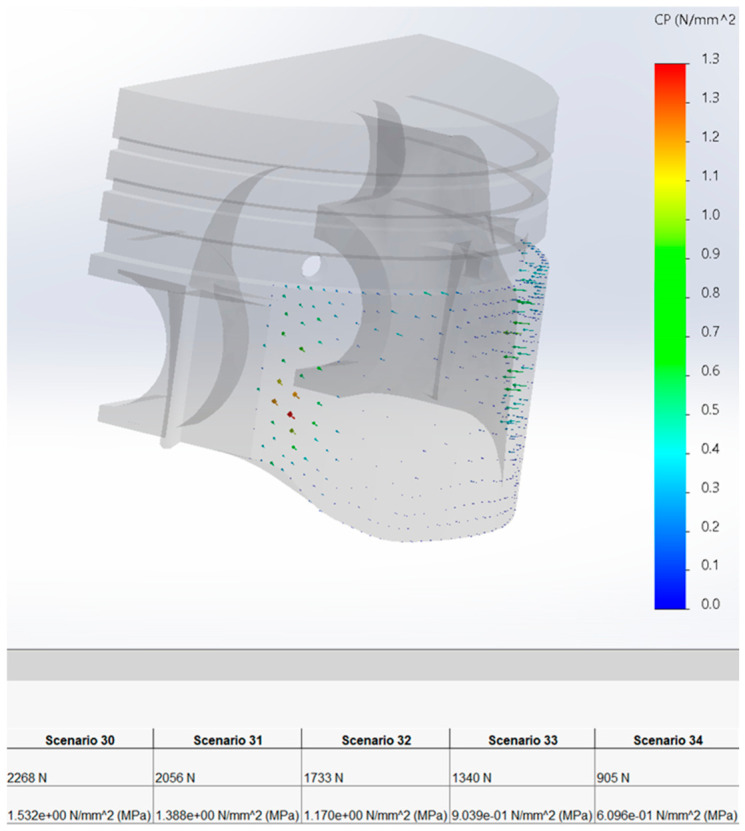
Design study of different normal forces applied and average contact pressure of the piston skirt.

The contact pressure distribution along the piston skirt was obtained through a parametric finite element design study under variable loading conditions corresponding to the engine cycle (Figure 6). For each discrete crank angle position (evaluated at 10° intervals), the normal force) *N*(φ) was applied, and the resulting contact pressure field was computed.

To ensure consistent comparison and applicability within the Archard wear model, the contact pressure *p* was defined as the area-averaged value over the active contact region of the piston skirt, rather than using local peak values. This approach provides a representative equivalent pressure corresponding to each loading step.

The results show that the contact pressure is not uniformly distributed but varies significantly with crank angle. Peak contact pressures occur in specific regions of the cycle, corresponding to maximum lateral forces acting on the piston due to combined gas and inertial loads.

Both positive and negative values of the normal force were considered in the analysis, as they correspond to contact on opposite sides of the piston skirt during the engine cycle. The positive values of the normal force were used to evaluate the contact pressure and wear on one side of the piston skirt, while the negative values were associated with contact on the opposite side and were used for the corresponding wear estimation. In this way, the alternating lateral loading of the piston was fully accounted for. The resulting pressure profile, expressed as a function of crank angle (or equivalent sliding distance), serves as the basis for subsequent wear calculations.

### 3.3. Equivalent Contact Parameter and Sliding Distance

**Figure 7:**
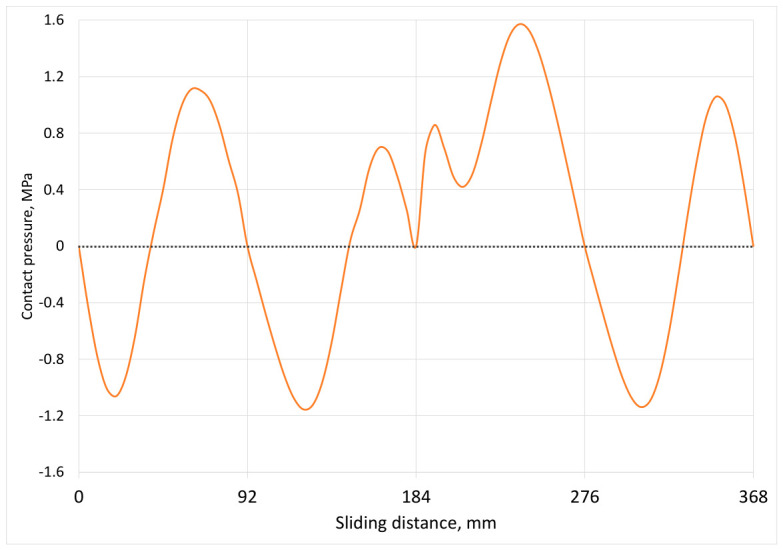
Contact pressure distribution through the piston sliding distance per one working cycle.

To account for the variable loading conditions during the engine cycle, the contact pressure was expressed as a function of the piston sliding distance. The crank angle was transformed into linear displacement based on piston kinematics (Figure 7).

Since the direction of the lateral force alternates during the cycle, the contact pressure values were separated into positive and negative components, corresponding to contact on the thrust and anti-thrust sides of the piston skirt, respectively.

Two equivalent contact parameters were determined by integrating the contact pressure over the sliding distance for each side independently:

(6) $$p_{eq}^{+}=\int_{cp>0}cp\;ds$$

(7) $$p_{eq}^{-}=\int_{cp<0}cp\;ds$$

where $p_{eq}^{+}$ and $p_{eq}^{-}$ represent the equivalent contact parameters for the two opposite sides of the piston skirt.

This approach allows the non-uniform and alternating loading conditions to be represented by physically meaningful parameters suitable for use in the Archard wear model, enabling independent evaluation of wear on both sides of the piston skirt.

Figure 7 clearly illustrates the alternating contact conditions acting on the piston skirt during one complete engine cycle. The positive and negative regions of the contact pressure distribution correspond to the periodic transfer of lateral loading between the thrust and anti-thrust sides of the piston. This behavior is characteristic of reciprocating piston motion and is essential for realistic wear evaluation, since the two sides of the piston skirt operate under different contact conditions during the cycle.

### 3.4. Wear Depth Estimation

The wear depth of the piston skirt was calculated using Equation (1), incorporating the experimentally determined wear coefficient *K*, the numerically obtained contact pressure, and the calculated sliding distance.

The results show that the wear depth varies significantly between the investigated materials. The lowest wear depth was observed for material M1 (13.94 nm for the thrust and 9.12 nm for the anti-thrust sides), while the highest value was obtained for M5 (27.61 nm for the thrust and 18.06 nm for the anti-thrust sides). The calculated wear depth for M3 is 18.27 nm for the thrust and 11.95 nm for the anti-thrust sides. It should be noted that the wear depth values presented in the study represent numerical estimations obtained using the Archard wear model combined with experimentally determined wear coefficients and finite element contact pressure analysis. Direct experimental measurement of piston skirt wear depth under real engine-cycle loading conditions was not performed within the scope of the present investigation.

Despite having the highest hardness, material M5 exhibits the largest wear, which confirms that hardness alone is not a sufficient indicator of wear resistance under the investigated conditions.

The obtained results demonstrate that the wear behavior of piston materials is governed by a complex interaction between mechanical properties, contact conditions, and tribological characteristics. The proposed methodology, combining experimental determination of the wear coefficient with numerical evaluation of contact pressure, provides a reliable approach for comparative assessment of material performance under realistic engine operating conditions.

These findings indicate that hardness alone cannot be used as a reliable indicator of wear resistance in hypereutectic Al–Si alloys. The increased wear observed for material M5 suggests that microstructural factors, such as silicon particle morphology and distribution, play a dominant role in determining tribological performance.

**Figure 8:**
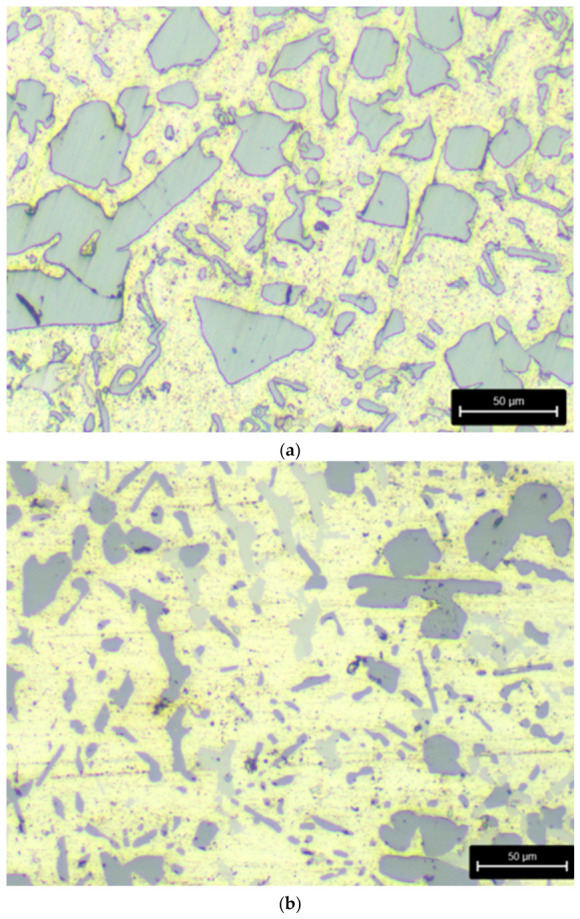

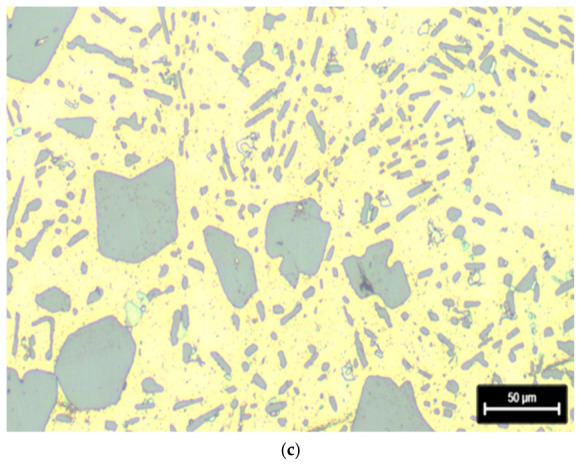
Optical microstructures of the investigated Al–Si alloys: (**a**) alloy M1; (**b**) alloy M3; (**c**) alloy M5.

To further interpret these results, an analysis of the microstructural characteristics of the alloys is required. As shown in [45], in alloy M1, the primary silicon is characterized by a relatively uniform morphology with an average size of about 20–25 μm, despite the presence of some larger particles (Figure 8a). The eutectic silicon is predominantly needle-like, which creates conditions for local stress concentrations; however, due to its relatively uniform distribution, it does not lead to intensive surface degradation.

Alloy M3 is characterized by a finer and spheroidized eutectic silicon phase (≈6–7 μm), which ensures a more uniform distribution of contact stresses and more stable tribological behavior (Figure 8b). Such a microstructure is associated with a lower tendency for crack initiation and improved wear performance [50,51]. Nevertheless, M3 contains intermetallic phases enriched in Cu, Co, and Cr, which increase hardness but may act as local stress concentrators, thereby limiting the expected improvement in wear resistance.

In alloy M5, a significant increase in the size of primary silicon (≈46 μm) is observed, along with a more complex and heterogeneous morphology of the eutectic phase (Figure 8c). The presence of coarse silicon crystals and hard intermetallic phases leads to a highly non-uniform stress distribution within the contact zone. Under sliding conditions, these structural features promote microcrack initiation, silicon particle pull-out, and the formation of abrasive third bodies, resulting in accelerated wear. Similar mechanisms have been widely reported for materials with coarse and brittle microstructures [51,52].

From a tribological perspective, these observations suggest a transition toward a more intensive abrasive wear mechanism in M5, whereas M1 and M3 exhibit more stable contact conditions with less pronounced surface degradation.

Although alloy M3 exhibits a more refined microstructure, the combined effect of microstructure and wear coefficient results in the lowest wear being observed for alloy M1. This behavior can be explained by the more balanced phase composition of M1, where silicon particles and intermetallic compounds are more uniformly distributed and exhibit lower susceptibility to brittle fracture. In addition, the lower concentration of hard and brittle intermetallic phases (such as Al_2_Cu and complex Al–Si–Fe–Ni–Cr phases) reduces the likelihood of microcrack initiation and particle detachment during sliding, leading to a more stable contact layer and limited development of abrasive wear.

These results indicate that the wear behavior of piston materials is governed by a complex interaction between mechanical properties, contact conditions, and microstructural characteristics. Despite the increase in hardness from 1363 MPa (M1) to 1677 MPa (M5), the wear coefficient increases significantly, resulting in greater wear depth. This clearly demonstrates that microstructural factors, such as the morphology and distribution of silicon particles and intermetallic phases, significantly influence determining the tribological behavior of hypereutectic Al–Si alloys.

The proposed methodology, combining experimental determination of the wear coefficient with numerical modeling of contact pressure, provides a reliable framework for linking microstructure to the actual wear mechanisms under operating conditions. Detailed quantitative stereological analysis of the investigated alloys has been previously reported in [46].

It should be noted that the experimental wear tests were conducted at room temperature under controlled lubrication conditions, whereas real piston operation occurs at significantly elevated temperatures. Under actual engine conditions, the temperature of the piston skirt may exceed 200 °C, which can influence both the mechanical and tribological behavior of Al–Si alloys. Elevated temperatures may lead to partial thermal softening of the aluminum matrix, modification of intermetallic phase stability, and changes in lubricant viscosity and lubrication regime. As a consequence, the absolute values of the wear coefficient and wear depth may differ from those obtained under laboratory conditions. Nevertheless, since all investigated materials were tested under identical experimental conditions and evaluated using the same numerical methodology, the comparative trends observed between the alloys remain representative and physically meaningful. Therefore, the proposed approach is considered suitable for comparative assessment of piston material wear behavior, while future investigations may include temperature-dependent tribological characterization for more accurate simulation of real engine operating conditions.

These results clearly demonstrate that increased hardness alone cannot be considered a reliable predictor of wear resistance in hypereutectic Al–Si piston alloys. The tribological performance is governed predominantly by microstructural stability, silicon particle morphology, phase distribution, and the tendency for brittle fracture and particle pull-out under reciprocating contact conditions.

### 3.5. Comparison with Previous Studies

Previous studies have widely applied Archard-based wear models for the investigation of tribological systems and reciprocating contacts. Xue et al. combined finite element contact analysis with Archard’s wear formulation for predicting wear evolution in self-lubricating bearings, demonstrating the importance of local contact pressure distribution for accurate wear estimation [53]. Similarly, Zabala et al. investigated piston ring–cylinder liner tribology under boundary lubrication conditions near top dead center and highlighted the strong influence of contact pressure, lubrication regime, and surface properties on wear behaviour [18]. Recent studies have also shown that the wear coefficient in Archard-type models cannot always be considered constant, as it may depend on contact conditions, deformation energy, and surface interactions [54]. Nevertheless, the Archard approach remains one of the most widely used engineering methods for comparative wear assessment due to its relatively simple implementation and good correlation with experimental observations [55].

Several investigations have focused on the tribological behaviour of piston materials and reciprocating sliding systems under engine-relevant operating conditions. Reciprocating wear testing of piston aluminum alloys under different loads and sliding velocities demonstrated that wear behaviour strongly depends on contact pressure, lubrication regime, and alloy microstructure [56]. Additional studies on piston skirt friction and wear systems emphasized the complexity of piston–cylinder interactions under variable loading conditions during the engine cycle [18,57]. Previous investigations of hypereutectic Al–Si alloys and coated piston systems mainly addressed seizure resistance, coating durability, and general tribological performance under reciprocating motion.

In contrast to previous studies mainly focused on coatings, steels, bearings, or conventional piston ring–liner contacts, the present study investigates the wear resistance of non-standardized Al–Si piston alloys by combining experimentally obtained tribological characteristics with finite element contact pressure analysis and engine-cycle-dependent loading conditions. The experimentally determined wear coefficient K varied from 12.14 × 10^−5^ for alloy M1 to 29.59 × 10^−5^ for alloy M5, while the calculated wear depth increased from 13.97 nm to 27.67 nm per engine cycle. Despite the increase in material hardness from 1363 MPa to 1677 MPa, the wear resistance deteriorated significantly. This tendency is consistent with previously reported observations for hypereutectic Al–Si alloys under reciprocating sliding conditions, where microstructural characteristics such as silicon particle morphology, intermetallic phase distribution, and structural heterogeneity were found to have a dominant influence on wear behaviour. Therefore, the main contribution of the present study lies in the integrated experimental–numerical methodology combining experimentally obtained wear characteristics with finite element evaluation of contact pressure under realistic piston loading conditions during the engine cycle. This approach enables a physically consistent comparative assessment of piston alloy wear behaviour under conditions closer to real engine operation.

## 4. Conclusions

This study investigated the wear behavior of three non-standardized hypereutectic Al–Si piston alloys (M1, M3, and M5) using an integrated experimental–numerical approach. Based on the obtained results, the following conclusions can be drawn:

- The wear resistance of the investigated alloys does not correlate directly with hardness. Although alloy M5 exhibited the highest hardness (1677 MPa), it also showed the highest experimentally determined wear coefficient (29.59 × 10^−5^) and the greatest predicted wear depth (27.67 nm per engine cycle). In contrast, alloy M1 demonstrated the lowest wear coefficient (12.14 × 10^−5^) and the smallest calculated wear depth (13.97 nm), indicating superior wear resistance under the investigated operating conditions.
- The experimentally determined wear coefficient K plays a decisive role in tribological performance, increasing significantly from M1 to M5 and directly governing the resulting wear depth.
- The contact pressure distribution along the piston skirt is highly non-uniform and varies throughout the engine cycle, leading to localized stress concentrations and increased sensitivity to material heterogeneity.
- Microstructural characteristics, including the size, morphology, and distribution of silicon particles and intermetallic phases, are the dominant factors controlling wear behavior. Coarse and heterogeneous structures promote crack initiation, particle pull-out, and abrasive wear.
- The integrated methodology, combining experimental determination of wear parameters with numerical evaluation of contact conditions, provides a physically consistent and reliable framework for comparative assessment of piston materials under realistic operating conditions.

From a practical engineering perspective, the obtained results indicate that increasing material hardness alone does not necessarily improve piston wear resistance under realistic reciprocating operating conditions. Although alloy M5 exhibited the highest hardness, it also demonstrated the greatest wear depth, indicating less favorable tribological behavior. In contrast, alloy M1 provided the best balance between hardness, microstructural characteristics, and wear resistance. Therefore, the results suggest that optimized microstructural stability and phase distribution are more important for piston durability than hardness alone. This finding is particularly relevant for the design and selection of advanced Al–Si piston alloys intended for highly loaded internal combustion engines.

Overall, the results demonstrate that optimization of microstructure and phase composition, rather than increasing hardness alone, is the key factor in improving the wear resistance of hypereutectic Al–Si piston alloys.

## Figures and Tables

**Table 1:** Summary of previous studies and identified research gaps.

Reference	Material Alloy	Main Focus	Methodology	Key Findings	Limitation
Singh et al. [39]	Al-7Si, WE43A	Thermal-structural piston analysis	FEA	Mg alloy shows better high-temp. behavior	No wear modelling
Wakshume & Sufe [40]	Al2618, Al4032, Ti-6Al-4V	Combined thermo-mechanical loading	ANSYS 19.2 FEA	Al2618 gives the lowest deformation	No tribological validation
Chandra et al. [26]	Novel Al-Si alloy	Mechanical and tribological behavior	Wear testing + casting study	Improved wear resistance with squeeze casting	Focused on rings, not piston alloys
Yang et al. [28]	Al-Si-Cu-Ni-Mg	High-temperature piston alloy phases	Microstructure analysis	Cu affects Ni-rich intermetallic evolution	Not focused on Al–Si piston alloys
Singhal et al. [25]	Al-Si AMCs	Wear behavior review	Literature review	Reinforcements improve wear resistance	

**Table 2:** Material properties.

Parameter	M1	M3	M5
Elastic Modulus, E [MPa]	91,000	93,000	92,000
Poisson’s Ratio, ν [-]	0.33	0.32	0.32
Density, ρ [kg/m^3^]	2685	2730	2710
Yield Strength, Rp_0.2_ [MPa]	184	181	204
Tensile Strength, Rm [MPa]	193	190	215

**Table 3:** Chemical Composition of the Investigated Alloys [45].

No.	Alloy	Si	Cu	Cr	Ni	Co	Mo	Fe	Al
M1	AlSi25Cu4Cr	24.01	3.73	0.633	0.752	-	0.114	0.298	rest
M3	AlSi25Cu5Cr	25.14	4.05	0.810	-	0.595	0.05	0.5	rest
M5	AlSi25Cu5Cr	25.31	4.32	0.528	0.005	-	-	0.122	rest

**Table 4:** Test parameters.

Parameter	M1
Normal load	20 N
Nominal contact area	7.07 mm^2^
Nominal contact pressure	2.83 × 10^4^ N/cm^2^
Sliding speed	6.8 cm/s
Friction time	15 min
Friction path	58.8 m
Ambient temperature	23 °C
Mass loss	
M1	0.3 mg
M3	0.4 mg
M5	0.6 mg
Material density	
M1	2685 kg/m^3^
M3	2730 kg/m^3^
M5	2710 kg/m^3^

**Table 5:** Material properties (H, K).

Parameter	M1	M3	M5
Hardnes HV5/10, kgf/mm^2^	139	152	171
Hardnes HV5/10, MPa^2^	1363	1491	1677
K, -	12.14 × 10^−5^	17.405 × 10^−5^	29.59 × 10^−5^

## Data Availability

The original contributions presented in this study are included in the article. Further inquiries can be directed to the corresponding authors.

## Abbreviations

The following abbreviations are used in this manuscript:

ICE	Internal combustion engine
Al–Si	Aluminum–silicon
HV	Vickers hardness
ISO	International organization for standardization
FEA	Finite element analysis
3D	Three-dimensional
XRD	X-ray diffraction
L	Liter s
